# Free-Living Physical Activity Measured With a Wearable Device Is Associated With Larger Hippocampus Volume and Greater Functional Connectivity in Healthy Older Adults: An Observational, Cross-Sectional Study in Northern Portugal

**DOI:** 10.3389/fnagi.2021.729060

**Published:** 2021-11-30

**Authors:** Célia Domingos, Maria Picó-Pérez, Ricardo Magalhães, Mariana Moreira, Nuno Sousa, José Miguel Pêgo, Nadine Correia Santos

**Affiliations:** ^1^Life and Health Sciences Research Institute (ICVS), School of Medicine, University of Minho, Braga, Portugal; ^2^ICVS/3B’s, PT Government Associate Laboratory, Braga, Portugal; ^3^iCognitus4ALL – IT Solutions, Braga, Portugal; ^4^Clinical Academic Center – Braga (2CA-B), Braga, Portugal; ^5^NeuroSpin, CEA, CNRS, Paris-Saclay University, Gif-sur-Yvette, France; ^6^ENCONTRAR+SE—Association for the Promotion of Mental Health, Porto, Portugal; ^7^Associação Centro de Medicina P5 (P5), School of Medicine, University of Minho, Braga, Portugal

**Keywords:** physical activity, elderly, brain health, neuroimaging, accelerometry

## Abstract

Several studies using neuroimaging techniques have established a positive relationship between physical activity (PA) and brain structure and function in older populations. However, the use of subjective measures of PA and the lack of multimodal neuroimaging approaches have limited the understanding of this association. This study aims to explore the associations between PA and brain structure and function by objectively evaluating PA. Community-dwelling cognitively healthy older adults (without diagnosed cognitive, neurological or degenerative disease) were recruited from local health centers and local gyms. In a cross-sectional design, participants were evaluated regarding cognitive, clinical, anthropometric, physical performance, and lifestyle characteristics. A 3 T magnetic resonance imaging (MRI) was performed for structural and functional brain measures. PA time and level was assessed via a Xiaomi Mi Band 2^®^ worn for 15 consecutive days. Participants (*n* = 110, after inclusion/exclusion criteria and completion of all evaluations) were 58 females (56%), with an average age of 68.42 years old (*SD* = 3.12), most were active. Multiple regression analysis revealed that higher time spent in vigorous PA associated with larger left parahippocampal gyrus and right hippocampus volumes. Furthermore, the analysis of the functional connectome indicated a greater functional connectivity (FC) between the frontal gyrus, cingulate gyrus, occipital inferior lobe for light, moderate, and total PA time, and sedentary time associated with lower FC in the same networks. Overall, the structural and functional findings may provide evidence on the relevant association between PA and brain health in aging.

## Introduction

Due to the global rise in life expectancy, the older population is increasing worldwide (Prakash et al., 2011; Boraxbekk et al., 2016). This has led to a pressing need to understand patterns of both healthy and unhealthy aging processes. Normal aging is associated with changes in brain structure and function, with progressive brain atrophy, and age-related cognitive decline (Ho et al., 2011; Zlatar et al., 2013, 2015; Soares et al., 2014; Lee et al., 2016; Marques et al., 2016). In particular, the reductions in brain volume are most pronounced in prefrontal, temporal, and parietal gray matter (Zlatar et al., 2015). Furthermore, aging is the most important risk factor for the development of dementia (Kim et al., 2016), which in turn is one of the most pressing health issues due to the enormous costs from morbidity, mortality, and loss of independence (van der Flier and Scheltens, 2005; Wattmo et al., 2014; Voss et al., 2016). Therefore, healthy brain aging is an essential component of overall well-being (Joo et al., 2016), with current public health goals encompassing the identification of mechanisms that contribute to brain health, protecting from structural and functional declines (Floel et al., 2010; Prakash et al., 2011; Boraxbekk et al., 2016).

Emerging evidence over the past decade has suggested the beneficial effects of physical activity (PA) on brain health and function, particularly in aging individuals (Floel et al., 2010; Bugg and Head, 2011; Zlatar et al., 2015). Research indicates that PA confers reduced risk for mild cognitive impairment and dementia and has beneficial effects in maintaining cognitive function in older adults (Voss et al., 2016; Siddarth et al., 2018). Additionally, multiple neuroimaging studies have shown that PA is predictive of larger hippocampal volume in healthy elderly individuals (Prakash et al., 2011; Kleemeyer et al., 2016; Arenaza-Urquijo et al., 2017). Furthermore, it contributes to greater connectivity between the hippocampus and the anterior cingulate cortex (Boraxbekk et al., 2016) and positively correlates with higher gray matter volume in the prefrontal cortex in older adults (Erickson et al., 2014; Sexton et al., 2016; Yamamoto et al., 2017). PA has also been associated with superior white matter (WM) integrity using diffusion tensor imaging in descriptive and interventional studies (Tian et al., 2015), and with greater WM volume, reduced severity of WM lesions, and improvements in WM microstructure (Tian et al., 2015; Sexton et al., 2016). The brain network connectivity also appears to be highly influenced by PA (Prakash et al., 2011). Voss et al. (2016) mentioned that cardiorespiratory fitness has a positive relationship with the functional connectivity (FC) of several cortical networks associated with age-related decline, with the strongest effect in the default mode network (DMN). Also, Boraxbekk et al. (2016) observed higher PA associated with stronger connectivity in the posterior DMN. Recently, Domingos et al. (2020) systematically review the relationship between PA and brain structure and function in older adults. This study provided compelling evidence that PA is associated with larger brain volumes (less brain atrophy), specifically in brain regions vulnerable to dementia, including the hippocampus, temporal, and frontal regions, and greater task-relevant activity in brain areas recruited in executive function and memory tasks (Domingos et al., 2021b).

In context, PA is a promising non-pharmacological interventional approach for promoting the health of the aging brain (Benedict et al., 2013; Sexton et al., 2016; Bittner et al., 2019) since it is a cost-effective, safe and accessible intervention, and can reduce the need for (costly) drug treatments with negative side effects (Voss et al., 2016). Despite evidence for associations between PA and brain health, this relationship is not fully established due to study limitations (Tian et al., 2015; Oberlin et al., 2016; Sexton et al., 2016). Most studies have relatively small sample sizes, are mainly based on single brain biomarkers, and/or employ subjective measures of PA (Boraxbekk et al., 2016; Oberlin et al., 2016; Yamamoto et al., 2017). Indeed, self-report questionnaires have been used to measure PA, with limited validity in measuring activities of daily living, including the limitations associated with a self-report activity. In contrast, wearable sensors can provide an objective and precise assessment of everyday activities and may allow the identification of specific PA patterns (Erickson et al., 2014; Schwenk et al., 2015) across time and populations. These sensors include tools to determine the PA intensity and duration without the need of intervention from participants to self-report their activities (Erickson et al., 2014). Furthermore, wearable systems are being increasingly cited as having great potential to improve the PA levels in the population, including older adults. However, more research must be done to fully understand the potential of this wearable technology as health interventions promoting behavioral changes (Mercer and Li, 2016).

The current study goes beyond previous works, by combining a multimodal imaging approach with an objective measurement of daily PA to explore brain health in an aging cohort and addressing some of the previous limitations. Specifically, in a cohort of cognitively healthy older adults, the study aim is to explore the association between PA dimensions and brain volume and function.

## Materials and Methods

### Participants

A convenience sample of 120 community-dwelling older adults was recruited from local healthcare centers and local gyms in the municipality of Braga, in Northern Portugal. The eligibility criteria were: (1) age comprised between 65–75 years of age; (2) capacity to understand the informed consent; (3) ability to attend the MRI session (e.g., without metallic implants, pacemakers, claustrophobia); (4) ability to wear Xiaomi Mi Band 2^®^ for fifteen consecutive days; (5) free of diagnosed neuropsychiatric, or neurodegenerative disorders; (6) adequate visual, auditory, and fine motor skills; and (7) not having any incapacity that limited independent walking. From the initial population, a final sample of 110 participants met all inclusion criteria and completed the study assessment sessions.

Data collected from this set of participants were also included in previous studies that evaluated the user experience of older adults with wearable devices (Domingos et al., 2020, 2021a).

### Data Collection and Instruments

**FIGURE 1:**
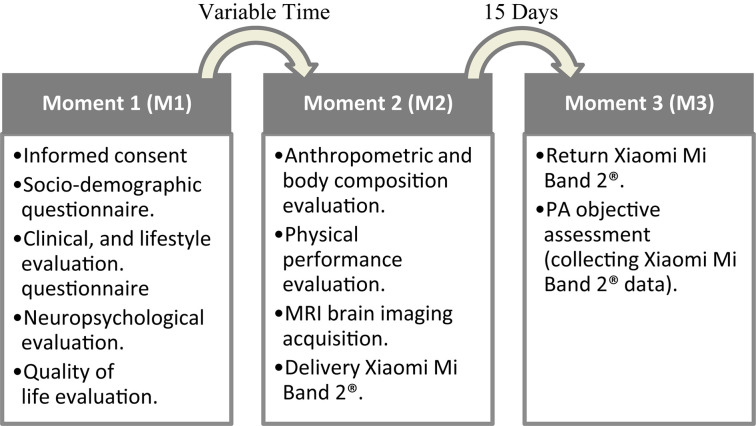
Flow diagram of the evaluation moments.

The participant’s characterization was performed through: (1) a structured questionnaire-based interview to assess socio-demographic, clinical, and lifestyle information; (2) a neuropsychological and quality of life evaluation; (3) an anthropometric and body composition evaluation; and (4) physical performance evaluation. Detailed information regarding the study evaluation moments is present in Figure 1. Based on the assessment type, measures were assessed always by the same attributed team member/researcher, following a study manual and/or established practices. Participants were instructed not to change their habitual daily living habits/routines.

#### Clinical and Lifestyle Characteristics

Clinical measures included current medication, clinical history of stroke, cardiac pathology, diabetes (diabetes mellitus type I or II), dyslipidemia, hypertension, psychiatric disorders, neurological disorders, chronic kidney disease, cancer, musculoskeletal disorders, digestive diseases, and chronic obstructive pulmonary disease. The variables were categorized as absence or presence of chronic diseases. Polypharmacy was defined as the use of three or more medications daily (Masnoon et al., 2017). The measurement of right brachial blood pressure (BP) was performed with the subject in a seated position after 5 min of resting time. Three readings, at 2-min intervals, were taken, and the mean was used for analysis.

For lifestyle measures, alcohol consumption and smoking habits (non-smoker, former smoker, and smoker) were considered. Heavy smokers were classified as smoking 25 or more cigarettes a day (Wilson et al., 1992) and excess alcohol consumption was considered more than 4 drinks per day for men and more than 3 drinks a day for women (National Institute of Alcohol Abuse and Alcoholism).

Comorbidity Index was calculated using the number of chronic conditions, polypharmacy, and lifestyle-related risk factors such as drink and smoking habits.

#### Anthropometric Characterization

The anthropometric evaluation included weight (Tanita BF 350 Body Composition Analyzer), height (Seca 217 Stadiometer), waist (WC), and hip circumferences (HP) (Ergonomic Measuring Tape). Body mass index (BMI) was obtained as weight (kg)/height (m2) and classified as underweight, normal, overweight, and obese (respectively, BMI: [0–18.5], [18.6–24.9], [25.0–29.9], and [30.0+]) (World Health Organization [WHO], 2011). The waist-to-hip ratio (WHR) was calculated by dividing the WC (cm) by HP (cm) circumference (World Health Organization [WHO], 2011). WHR above 0.90 for males and above 0.85 for females was classified as a substantially increased risk of metabolic complications (World Health Organization [WHO], 2011).

#### Body Composition

Body composition was assessed by bioelectrical impedance using a Maltron BioScan 920-II Multi-frequency Analyzer. The analysis was performed according to manufacturer instructions. The optimal cut-off values for body fat (FAT) percentage were defined as ≤ 42% for women and ≤ 30% for men (Pérez-Ros et al., 2020).

#### Physical Performance

The assessment of physical performance included gait speed (6-m walking test) and handgrip strength (Jamar Hand Dynamometer). In older individuals, the cut-off point for slow gait speed was defined as walking less than 1 m/s, which identifies persons at high risk of health-related outcomes in well-functioning (Cesari et al., 2005). The normative data for handgrip strength ranges from 22.5 to 25.6 kg for women and 36.2 to 41.7 kg for men (Bohannon et al., 2006).

Functional status was evaluated using the Lawton Instrumental Activities of Daily Living (IADL) Scale to assess independent living skills (Lawton and Brody, 1969), Katz Activities of Daily Living (ADL) Scale to assess six primary and psychosocial functions (bathing, dressing, going to the toilet, transferring, feeding, and continence) (Katz et al., 1963), and Tinetti-test to assess the gait and balance (Tinetti, 1986).

#### Neuropsychological Evaluation

A trained psychologist applied and scored all neuropsychological tests aiming to assess memory, executive function, general cognition, and mood. The Mini-Mental State Examination (MMSE) was used to assess the global cognitive profile (Guerreiro et al., 1994). The following cut-off scores for “cognitive impairment” were used: individuals with no education, <15 points; 1–11 years of school completed, <22 points; and >11 years of school completed, <27 points (Santana et al., 2016). The Selective Reminding Test (SRT) was used for verbal learning and memory (Buschke et al., 1995). The Digits Span Test (DSST) was used to test working memory and attention (Ryan and Lopez, 2001). The Stroop test assessed selective attention, cognitive flexibility, and response inhibition (Spreen and Strauss, 1998). To evaluate the level of interference when the name of a color is written in a different color ink the Golden index was used, with higher score means indicating decreased Stroop interference (Lansbergen et al., 2007). Verbal fluency was evaluated through the Controlled Oral Word Association F-A-S (FAS) test (Lezak et al., 2004). Finally, the Geriatric Depression Scale (GDS) was used to characterize depressive mood (Yesavage et al., 1982). GDS scores range from 0 to 30, representing the total number of depressive symptoms, with values > 11 indicating the presence of depressive symptomatology (Pocinho et al., 2009).

#### Quality of Life Assessment

Health-related quality of life was assessed using version 2.0 of the Portuguese 36-Item Short-Form Health Survey (SF-36) (Ferreira, 2003). The SF-36 is probably the most widely used generic health status measure used in clinical research, health policy evaluations, and general population surveys (Ware and Sherbourne, 1992; Fryback et al., 2007). The survey consists of 36 items that assess eight health concepts: physical functioning (PF), role physical (RP), bodily pain (BP), general health (GH), vitality (VT), social functioning (SF), role emotional (RE), and mental health (MH). These domains may be reduced into the physical component summary (PCS) and the mental component summary (MCS). Items are scored by a Likert scale. All items of SF-36 are used to score the eight domains, except for item 2, which refers to a self-report of health transition. Each item contributes to only one domain. The scores range from 0 (worst health) to 100 (best health) (Ware et al., 1994; Ware, 2000).

#### Xiaomi Mi Band 2^®^

The wearable activity tracker selection was based on a review of the characteristics of several different commercially available devices on the market (El-Amrawy and Nounou, 2015; Liang et al., 2018; Xie et al., 2018). The selection criteria included the popularity in the wearable healthcare devices market, availability, continuous monitoring of PA without a smartphone, price, battery life, various sensors data captured, and ability to export data (Domingos et al., 2021a). The Xiaomi Mi Band 2^®^ was selected because it offers the best price-quality ratio, has an estimated battery life of almost 30 days, is ergonomic, accessible, and easy to operate. The device measures steps, intensity, energy expenditure, and distance traveled (El-Amrawy and Nounou, 2015; Puri et al., 2017; Xie et al., 2018; Mičková et al., 2019). Additionally, Xiaomi Mi Band 2^®^ demonstrated its high measurement accuracy (96.56%) and the relatively low variation coefficient (CV = 5.81) (Xie et al., 2018).

Participants wore a Xiaomi Mi Band 2^®^ for 15 consecutive days while performing their normal daily activities. The data was exported in SQLite format using a Master for Mi Band app and then converted in CSV format.

##### Data Reduction

Microsoft Excel was used to perform data reduction and cleaning. Seven consecutive valid days of data were collected from the total wear time. For the analysis, we excluded the first day of records to ensure a complete day in each data collection period. Regarding data validation, each participant must have a minimum of five valid days, including at least one weekend day (Nicaise et al., 2014; O’Neill et al., 2017), wherein a valid day consisted of at least 10 h of records. An invalid hour was defined as more than 30 consecutive minutes with zero activity (Troiano et al., 2008; Ferrari et al., 2020). The PA intensities were defined using the following intensity-specific cut-points: sedentary 0–19 steps/min; light intensity: 60–99 steps/min; moderate intensity: 100–119 steps/min; and vigorous-intensity: ≥120 steps/min (Tudor-Locke et al., 2011, 2018).

### Magnetic Resonance Imaging Brain Imaging Acquisition

Sessions of MRI scanning were performed using a Siemens Verio 3T (Siemens, Erlangen, Germany) at Hospital de Braga (Braga, Portugal), using a 32-channel head antenna for reception. The scanning protocol included an anatomical acquisition with a T1-weighted (T1w) sagittal Magnetization-Prepared rapid acquisition with gradient echo (MPRAGE, TE/TR = 2420/4.12 ms, FA = 9°, 1 mm^3^ isometric voxel size, Field-of-View = 176 × 256 × 256 mm^3^). For the resting-state functional magnetic resonance imaging (rs-fMRI) acquisition a multi-band Echo Planar Imaging sequence was used, CMRR EPI 2D (R2016A, Center for Magnetic Resonance Research, University of Minnesota, Minnesota, United States) (Feinberg et al., 2010; Moeller et al., 2010; Xu et al., 2013) sensitive to fluctuations in the BOLD contrast (TR/TE = 1000/27 ms, FA = 62°, 2 mm^3^ isometric voxel size, 64 axial slices over an in the plane matrix of 100 × 100). The rs-fMRI acquisition had a duration of seven and a half minutes, during which subjects were asked to remain relaxed and with their eyes closed.

### Preprocessing of Magnetic Resonance Imaging Data

Preprocessing was performed using fMRIPrep 1.4.1 proposed by Esteban et al. (2019) (RRID:SCR_016216), which is based on Nipype 1.2.0 (RRID:SCR_002502) (Gorgolewski et al., 2011).

#### Anatomical Data Preprocessing

T1w image was corrected for intensity non-uniformity (INU) with N4BiasFieldCorrection (Tustison et al., 2010), distributed with ANTs 2.2.0 RRID:SCR_004757 (Avants et al., 2008), and used as T1w-reference throughout the workflow. The T1w-reference was then skull-stripped with a Nipype implementation of the antsBrainExtraction.sh workflow (from ANTs), with OASIS30ANTs as target template. Brain tissue segmentation of cerebrospinal fluid (CSF), white-matter (WM) and gray-matter (GM) was performed on the brain-extracted T1w using fast (FSL 5.0.9, RRID:SCR_002823) (Zhang et al., 2001). Brain surfaces were reconstructed using recon-all (FreeSurfer 6.0.1, RRID:SCR_001847) (Dale et al., 1999), and the brain mask estimated previously was refined with a custom variation of the method to reconcile ANTs-derived and FreeSurfer-derived segmentations of the cortical gray-matter of Mindboggle (RRID:SCR_002438) (Klein et al., 2017). Volume-based spatial normalization to standard space (ICBM 152 Non-linear Asymmetrical template version 2009c (RRID:SCR_008796; TemplateFlow ID: MNI152NLin2009cAsym) (Fonov et al., 2009) was performed through non-linear registration with antsRegistration (ANTs 2.2.0), using brain-extracted versions of both T1w reference and the T1w template.

#### Resting-State Data Preprocessing

For each subject, the following preprocessing steps were performed: First, a reference volume and its skull-stripped version were generated using a custom methodology of fMRIPrep. A deformation field to correct for susceptibility distortions was estimated based on a field map that was co-registered to the BOLD reference, using a custom workflow of fMRIPrep derived from D. Greve’s epidewarp.fsl script and further improvements of HCP Pipelines (Glasser et al., 2013). Based on the estimated susceptibility distortion, an unwarped BOLD reference was calculated for a more accurate co-registration with the anatomical reference. The BOLD reference was then co-registered to the T1w reference using bbregister (FreeSurfer) which implements boundary-based registration (Greve and Fischl, 2009). Co-registration was configured with nine degrees of freedom to account for distortions remaining in the BOLD reference. Head-motion parameters with respect to the BOLD reference (transformation matrices, and six corresponding rotation and translation parameters) were estimated before any spatiotemporal filtering using mcflirt (FSL 5.0.9) (Jenkinson et al., 2002). BOLD runs were slice-time corrected using 3dTshift from AFNI 20160207 (RRID:SCR_005927) (Cox, 1996). The BOLD time-series (slice-time corrected) were resampled onto their original, native space by applying a single, composite transform to correct for head-motion and susceptibility distortions, and finally resampled into MNI152NLin2009cAsym space. Several confounding time-series were calculated based on the preprocessed BOLD: framewise displacement (FD), DVARS (rate of change of BOLD signal across the entire brain at each frame of data), and three region-wise global signals. FD and DVARS are calculated for each functional run, both using their implementations in Nipype [following the definitions by Power et al. (2014)]. The three global signals were extracted within the CSF, the WM, and the whole-brain masks. Additionally, a set of physiological regressors were extracted to allow for component-based noise correction (CompCor) (Behzadi et al., 2007). Principal components were estimated after high-pass filtering the preprocessed BOLD time-series (using a discrete cosine filter with 128 s cut-off) for the anatomical variant (aCompCor). A mask covering the subcortical regions was obtained by heavily eroding the brain mask, which ensures it does not include cortical GM regions. For aCompCor, components were calculated within the intersection of the aforementioned mask and the union of CSF and WM masks calculated in T1w space, after their projection to the native space of each functional run (using the inverse BOLD-to-T1w transformation). Components were calculated separately within the WM and CSF masks. For each CompCor decomposition, the k components with the largest singular values were retained, such that the retained components’ time series were sufficient to explain 50 percent of variance across the nuisance mask (CSF, WM, combined, or temporal). The remaining components were dropped from consideration. The mean CSF and WM signals, as well as the first 6 aCompCor components, the FD and the DVARS were regressed as confounds from the BOLD data using fslregfilt. Excessive movement was considered with a mean FD >0.25. Thus, subjects that exceeded this threshold were excluded. Finally, fslmaths was used to spatially smooth (with a FWHM kernel of 6 mm) and band-pass filter (between 0.01 and 0.08 Hz) the resulting time-series.

### Voxel-Based Morphometry

FMRIB software library (FSL)-voxel-based morphometry (VBM) was used to analyze structural data (Douaud et al., 2007) an optimized VBM protocol (Good et al., 2001) conducted with FSL (Smith et al., 2004). First, structural images (which were already brain-extracted and gray matter-segmented by FMRIPrep) were registered to the MNI 152 standard space using non-linear registration (Andersson et al., 2007). The images obtained were averaged and flipped along the *x*-axis to create a left-right symmetric, study-specific GM template. Second, all native GM images were non-linearly registered to this study-specific template and “modulated” to correct for local expansion (or contraction) due to the non-linear component of the spatial transformation. The modulated GMimages were subsequently smoothed with an isotropic Gaussian kernel with a sigma of 3 mm.

### Network Based Statistics

Analysis of the functional connectome and correlation with variables of interest was carried using Network Based Statistics (NBS) (Zalesky et al., 2010). Time-series were extracted for each subject using the Shen atlas (Shen et al., 2013) with a total of 264 regions-of-interest covering the entire brain. Matrices of FC were calculated through the fisher-*z* normalized Pearson correlation of each pair of time-series, producing a 264-by-264 matrix of FC per-subject. Associations between the variables of interest and networks of FC were calculated using the NBS methodology. NBS produces non-parametric FWE corrected results in two steps: first by calculating the hypothesis at each point in the matrix and detecting connected networks of results significant according to a user specified primary threshold; and second by calculating the significance of that network through permutation testing and comparing the extent of the network at a FWE-corrected *p*-value of 0.05. Because different primary thresholds can yield networks of different nature it is recommended to explore a range of values. In this work we tested N significance values between 0.005 and 0.00001. Visualization of the significant results was produced using BrainNet Viewer (Xia et al., 2013). The scripts used to perform the analysis and produce all statistics are freely available online^1^.

### Statistical Analysis

The sample size was calculated using GPower with an effect size of 0.32 (Lautenschlager et al., 2012; Hogan et al., 2013; Gajewski and Falkenstein, 2016). Descriptive analysis was performed for all variables. Quantitative variables were expressed as mean and standard deviation (SD), and the results for the categorical variables were expressed as percentages and 95% confidence intervals. Normal data distribution was examined using the Shapiro–Wilk test, skewness, kurtosis, and histograms. Absolute values for skewness above 2.0 and kurtosis above 4.0 were considered as reference values for determining normality. A *p*-value below 0.05 for Shapiro–Wilk test indicates that the data significantly deviate from a normal distribution (Mishra et al., 2019).

Spearman correlations were used to assess associations between neuropsychological characteristics and PA components. The correlation coefficient was analyzed considering the Rule of Thumb (Hinkle et al., 2003).

To explore whether components of PA, including total PA, light, moderate, vigorous, and sedentary time, as measured cross-sectionally via the Xiaomi Mi Band 2^®^ for fifteen consecutive days, associated with brain volume and resting-state networks (NBS), all PA components were analyzed separately, after controlling for age, gender, MMSE, and GDS. Positive and negative correlations were tested for all variables. Regarding VBM analysis, threshold-free cluster enhancement (TFCE) was used to detect widespread significant differences and control the family wise error rate (FWE-R) at α = 0.05. Five thousand permutations were performed for each contrast. Moreover, the analysis was performed at the whole-brain level (using a gray matter mask), as well as using a hippocampus mask, due to the putative role of PA on this region (Prakash et al., 2011; Kleemeyer et al., 2016; Arenaza-Urquijo et al., 2017).

## Results

### Study Participants

**FIGURE 2:**
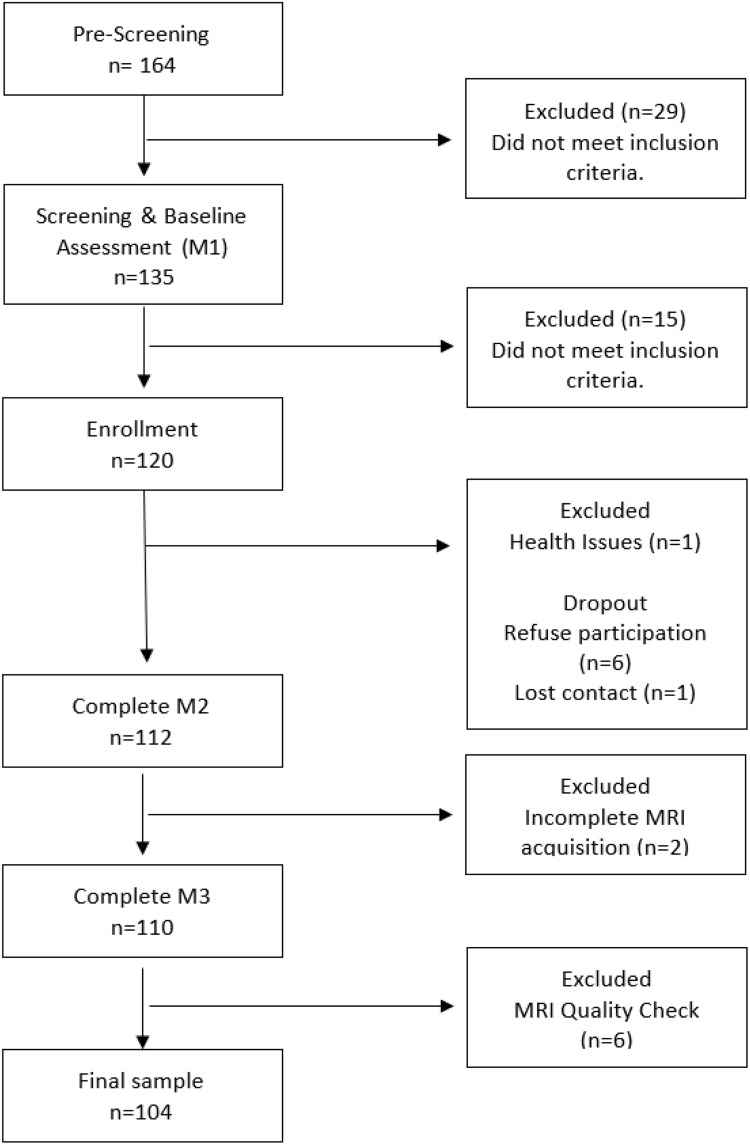
Flow diagram of participant screening and enrollment.

The number and flow of participants from screening through enrollment are displayed in Figure 2 and the general participant’s characteristics are summarized in Table 1. The older adults included in the final analysis had a mean age of 68.42 (SD ± 3.12), 56% were female, and the number of formal years of education was 7.75 (SD ± 5.28). Regarding the lifestyle habits, 9.6% (*n* = 10) were classified as heavy smokers, and 6.7% (*n* = 7) were considered as having an excess of alcohol consumption. In this sample, 23.1% (*n* = 24) were identified as having polypharmacy and 16.3% (*n* = 17) have a comorbidity index ≥ 5. The average BMI (28.83 ± 4.24) and the WHR ratio (0.95 ± 0.07) indicated that the older adults comprising the sample had a substantially increased risk of metabolic complications. The gait speed and handgrip strength were in the range of reference values, showing a good physical performance, and the functional status evaluation confirmed the independence in activities of daily living and performance of instrumental activities of daily living.

**TABLE 1:** General characteristics of the study participants (*n* = 104).

**Characteristics**	**N (%) or mean ± SD**
Gender, *n* (%female)	58 (55.8)
Age, years	68.42 ± 3.12
Education, years	7.75 ± 5.28
Systolic BP (mmHg)	130.95 ± 15.42
Diastolic BP (mmHg)	7.92 ± 11.87
HR	69.79 ± 10.81
**Smoking status**	
Current *n* (%)	10 (9.62)
Never *n* (%)	68 ± 65.38
Former *n* (%)	26 ± 25.00
Heavy smoker *n* (%)	10 ± 9.62
**Alcohol consumption**	
Alcohol drinker *n* (%)	75 ± 72.12
Excessive consumption *n* (%)	7 ± 6.73
**Polypharmacy, *n* (%)**	
<3	80 ± 7.92
≥3	24 ± 23.08
**Comorbidity Index**	
0	12 ± 11,54
≥5	17 ± 16.35
**Anthropometric**	
Weight, Kg	73.59 ± 13.27
Height, m^2^	159.58 ± 8.43
BMI, kg/m^2^	28.83 ± 4.24
WHR	0.95 ± 0.07
**Body composition**	
FAT,%	33.70 ± 7.83
FFM, %	66.30 ± 7.83
Muscle, Kg	23.61 ± 4.24
Malnutrition Index	0.66 ± 0.05
**Physical performance**	
ADL score	5.66 ± 0.51
IADL score	7.97 ± 0.16
Balance score	15.57 ± 0.73
Gait score	11.97 ± 0.22
Max Right Grip, Kg	30.85 ± 10.11
Max Left Grip, Kg	29.47 ± 9.66
Speed, m/s	64.91 ± 21.28

*BP, blood pressure; HR, heart rate, BMI, Body mass index; WC, waist circumference; HP, hip circumference; WHR, waist-to-hip ratio; FAT, Fat Mass; FFM, Free Fat Mass; Muscle, Muscle mass; ADL, Activities of Daily Living; IADL, Instrumental Activities of Daily Living.*

Regarding the neuropsychological assessment, all participants presented values above the cut-off scores. The mean score for MMSE was 26.96 (SD ± 1.98) and 6.09 (SD ± 4.56) for GDS. The cognitive variables are presented in Table 2. The SF-36 descriptive statistics for the eight scale domains and the two components (physical and mental health) are displayed in Table 3. The mean scores indicated a good health-related quality of life. The mean scores for PCS and MCS were 50.08 (SD ± 9.76) and 49.55 (SD ± 9.83), respectively. Concerning Xiaomi Mi Band 2^®^-measured PA, the results showed that the median time spent in sedentary activities was 1296.27 min.day^–1^ (SD 106.99), light-intensity activity was 40.06 min.day^–1^ (SD 57.54), moderate-intensity was 17.24 min.day^–1^ (SD 18.32), and vigorous-intensity was 9.31 (SD 16.56). The data show that participants accumulated more time a day in light-intensity activities (Table 4). Furthermore, 56% of participants were physically active (somewhat active, *n* = 27; active *n* = 16; highly active, *n* = 15) and 51% (*n* = 53) were found to meet the guidelines of a minimum of 8000 steps/day (Table 5).

**TABLE 2:** Neuropsychological characterization of the study participants (*n* = 104).

**Neuropsychologic characteristics**	**Mean ± SD**
MMSE	26.96 ± 1.98
GDS	6.09 ± 4.56
SRT-LTS	20.56 ± 13.34
SRT-CLTR	13.59 ± 12.37
SRT-DR	5,41 ± 2.59
Stroop-W	66.75 ± 19.72
Stroop-C	49.62 ± 10.65
Stroop-WC	26.11 ± 10.11
Stroop-golden	−2.01 ± 8.06
DSST score	32.54 ± 15.67
Digits Span Test	12.96 ± 3.72
COWAT FAS Admissible	21.96 ± 11.08

*MMSE, Mini Mental State Examination; GDS, Geriatric Depression Scale; SRT, Selective Reminding Test; LTS, long term storage; CLTR, consistent long-term retrieval; DR, delayed recall; Digits Span Test; DSST, Digit Symbol Substitution Test; w, word; c, color; wc, color-word; COWAT FAS, Controlled Oral Word Association Test.*

**TABLE 3:** Descriptive statistics for SF-36 dimensions (*n* = 104).

**SF-36 domain**	**Mean ± SD**
Physical functioning (PF)	86.88 ± 15.78
Role physical (RP)	83.95 ± 23.07
Bodily pain (BP)	44.47 ± 10.22
General health (GH)	73.56 ± 17.16
Vitality (VT)	86.18 ± 16.19
Social functioning (SF)	89.66 ± 17.72
Role emotional (RE)	94.87 ± 12.74
Mental health (MH)	89.57 ± 10.64
**F-36 component scores**	
Physical component summary (PCS)	50.08 ± 9.76
Mental component summary (MCS)	49.55 ± 9.83

**TABLE 4:** Descriptive PA data obtained from Xiaomi Mi Band 2^®^ (*n* = 104).

**Characteristics**	**Mean ± SD**
**Steps (steps.day^–1^)**	
Light PA	3180.82 ± 4651.32
Moderate PA	1883,34 ± 1988.94
Vigorous PA	1263.68 ± 2311.07
Total steps	9902.67 ± 8433.75
**Time (min.day^–1^)**	
Light PA	40.06 ± 57.54
Moderate PA	17.24 ± 18.32
Vigorous PA	9.31 ± 16.56
Total PA	143.73 ± 106.99
Sedentary time (min.day^–1^)	1296.27 ± 106.99

**TABLE 5:** Classification of older individuals according to PA Guidelines (*n* = 104).

**Characteristics**	***n* (%)**
**Classification**	
Sedentary	16 (15.4)
Low active	30 (28.8)
Somewhat active	27 (26.0)
Active	16 (15.4)
Highly active	15 ± 14.4
**PA recommendation, *n* (%)**	
Meeting PA	53 ± 51.0
Not meeting PA	51 ± 49.0

Concerning correlations between neuropsychological characteristics and PA components, a significant positive correlation (*r* = 0.196, *p* = 0.046) between moderate PA and DSST score was found (Supplementary Table 1), while no other correlations were significant for the other relationships tested.

Finally, Supplementary Figures 1, 2 show the distribution of PA components according to gender, presenting a similar distribution for both males and females.

### Voxel-Based Morphometry

**FIGURE 3:**
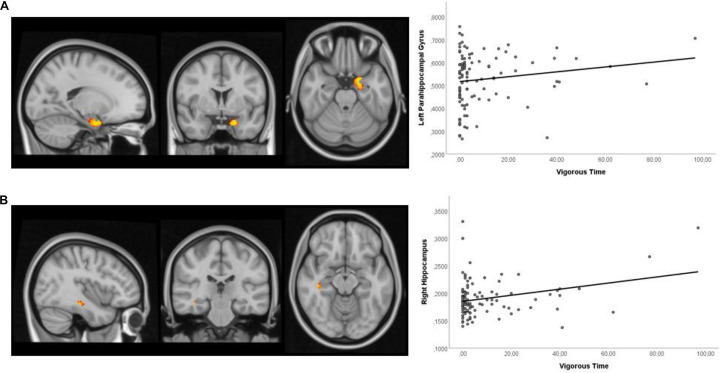
Sagittal, coronal, and axial view of the clusters and the scatter plot showing a significant positive correlation between vigorous time and: **(A)** left parahippocampal gyrus (–18, 2, –24) and **(B)** right hippocampus (40, –26, –14), after controlling for age, gender, age, MMSE, and GDS. All clusters illustrated were defined with a threshold at a *p*-value of 0.1.

Higher time spent vigorous PA was positively associated with hippocampal volumes when using the hippocampal mask (Figure 3). The significant peak voxels were found in the left parahippocampal gyrus and right hippocampus (Table 6).

**TABLE 6:** VBM results – Regression analysis of PA and brain volume (*n* = 104).

**Brain mask**	**Contrast**	**Effect direction**	**Ke**	**MNI Coordinates (x, y, z)**	**Region**	**Peak *t*-value**
Hippocampus	Vigorous time	+	114	–18, 2, –24	Left parahippocampal gyrus	3.42
		+	6	40, –26, –14	Right hippocampus	4.43

*Ke, Cluster extent; MNI, Montreal Neurological Institute.*

### Network Based Statistics

**FIGURE 4:**
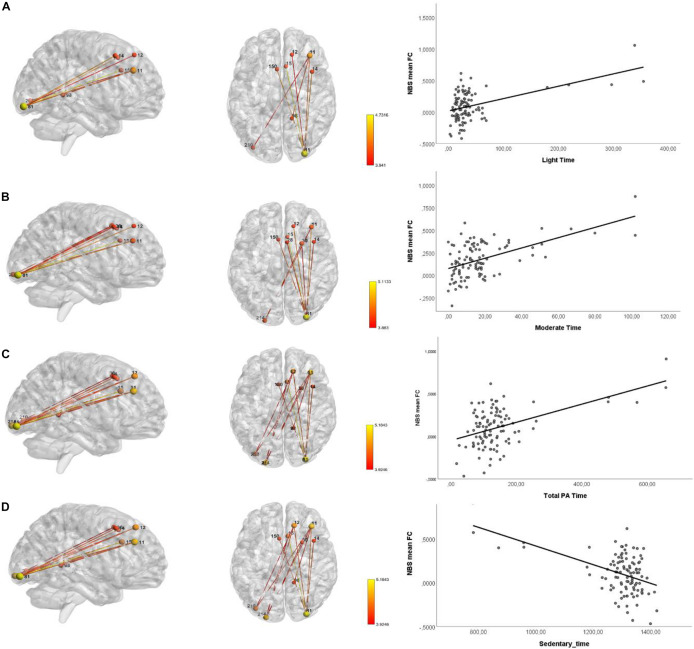
Networks of FC identified using NBS showing increased connectivity during rs-fMRI acquisition. Sagittal and axial view of the network with nodes and edges colored in red-yellow color scheme representing the statistical *t*-value and the scatter plot showing the relationships between the mean FC and: **(A)** light time; **(B)** moderate time; **(C)** total PA time; and **(D)** sedentary time; after controlling for age, gender, age, MMSE, and GDS.

Table 7 and Figure 4 show the networks identified using NBS with a significantly increased FC. The analysis revealed a significant increase in FC in a network involving nodes of the frontal cortex (superior gyrus #11, #12, frontal middle gyrus #14, and medial frontal gyrus #150), occipital inferior gyrus (#81 and #214), and cingulate gyrus (#15) was observed for light, moderate and total PA time, and for sedentary time. Moreover, the significant FC observed in the cerebellum limbic (parahippocampal gyrus #98) was specific for light and total PA time, and for sedentary time.

**TABLE 7:** Results of the functional connectomics analysis using NBS (*n* = 104).

**Variable**	**Threshold (t, p)**	**Effect direction**	***p-*value**	***R* ^2^**	**N. ° Nodes**	**N. ° Edges**
Light time	(3,8631, 1*10^–4^)	+	0.044	0.14	6	5
Moderate time	(3,8631, 1*10^–4^)	+	0.040	0.145	9	9
Moderate time	(4,055, 5*10^–5^)	+	0.044	0.159	6	5
Moderate time	(4,4807, 1*10^–5^)	+	0.018	0.171	4	3
Total PA time	(3,8631, 1*10^–4^)	+	0.034	0.141	10	12
Total PA time	(4,055, 5*10^–5^)	+	0.022	0.146	9	8
Sedentary time	(3,8631, 1*10^–4^)	–	0.031	0.1411	10	12
Sedentary time	(4,055, 5*10^–5^)	–	0.026	0.1461	9	8

## Discussion

Multiple neuroimaging studies have provided evidence that PA has protective effects on brain structure and function in older adults (Domingos et al., 2021b). However, the use of subjective measures of PA and the lack of a multimodal MRI approach have limited the understanding of this association. Therefore, in this study, we used objective PA data (obtained from a Xiaomi Mi Band 2) combined with neuroimaging acquisitions to test whether PA is related to the preservation of brain volume and better brain function in brain-healthy older adults (without neuropathological disease or dementia). The main findings from this study were as follows: (1) higher time spent in vigorous PA associated with larger volumes in left parahippocampal gyrus and right hippocampus; (2) light, moderate and total PA time associated with greater FC between the frontal gyrus (superior, middle, and medial), cingulate gyrus, occipital inferior lobe and parahippocampal gyrus, and (3) sedentary time associated with lower FC in the same networks.

The structural MRI results validated the findings from previous studies that show a consistent positive association between hippocampus and PA. Specifically, previous cross-sectional studies in older adults, using objective measures of PA, observed that a greater amount, duration, and frequency of total daily walking activity were associated with larger hippocampal volume (Varma et al., 2015), and larger hippocampal surface area (Varma et al., 2016) in women, but not men. However, these previous results were specific for low-intensity activities in contrast to our results that were specific for moderate and vigorous activities. The association between hippocampus volume and PA was also noticed in cross-sectional studies in older adults using self-report PA measures (Zlatar et al., 2015; Hashimoto et al., 2017). In addition to the hippocampus, previous cross-sectional studies using objective measures of PA have shown the association with a lower volume of WMH (Burzynska et al., 2014), higher fractional anisotropy in the temporal lobe (Burzynska et al., 2014), and greater inferior and anterior temporal lobe volumes (Dougherty et al., 2016). Regarding longitudinal studies, other authors observed that the maintenance of time spent walking predicted less reduction in hippocampal volume 13 years later (Best et al., 2017), and greater walking distances associated with greater GM in the hippocampus 9 years later (Erickson et al., 2010).

To summarize, the finding of a positive association between higher time spent in vigorous PA with larger hippocampal volume is of particular relevance, since hippocampal atrophy is associated with cognitive decline, memory impairment, and dementia, and is a well-established neuroimaging biomarker in the preclinical stages of Alzheimer’s disease (Varma et al., 2015; Arenaza-Urquijo et al., 2017; Hashimoto et al., 2017), and goes in hand with a recent systematic review suggesting that PA may also attenuate the age-related decline in brain regions vulnerable to dementia, including the hippocampus, temporal, and frontal regions (Domingos et al., 2021b).

Regarding brain FC, the present results suggest that higher levels of moderate and total PA time are positively associated with increased FC in areas responsible for cognitive functions, particularly in working memory (du Boisgueheneuc et al., 2006), executive attention (Japee et al., 2015) decision-related processes (Talati and Hirsch, 2005), visuospatial processing and episodic memory (Aminoff et al., 2013), regulation of motor function and processing emotions (Cera et al., 2019), and visual processing (Fan et al., 2020). Furthermore, sedentary behavior was associated with decreased FC in the same brain areas. These results are in line with a recent systematic review that suggests a positive relationship between PA and executive function and memory performance (Domingos et al., 2021b). Other cross-sectional studies in older adults have shown increased activation in BOLD response (famous > unfamiliar faces) in medial frontal gyrus/supplementary motor area, left precentral gyrus, middle/superior frontal gyrus, left middle/inferior temporal gyrus/angular gyrus/supramarginal gyrus/precuneus (Smith et al., 2011). A greater BOLD response in the right angular gyrus/lateral occipital cortex and left supramarginal gyrus (Zlatar et al., 2013), left hippocampus (Thielen et al., 2016) dorsolateral prefrontal cortex (Kimura et al., 2013), and greater FC between mPFC and left thalamus/right hippocampus and insula (Thielen et al., 2016), and greater FC between default-mode and amygdala/hippocampus/ventromedial prefrontal cortex and lower connectivity between default-mode and precentral gyrus/postcentral gyrus/supplementary motor cortex (Voss et al., 2016), was also observed.

This study adds to the literature by using objective measures of PA to determine its level and, thus, suppressing the limitation associated with self-report measures, in exploring the association between PA and brain function and structure in community-dwelling older individuals. The cohort was further well-characterized regarding clinical and lifestyle characteristics, anthropometric and body composition measures, physical performance, cognitive measures informative of broad cognitive domains, and quality of life assessment. Furthermore, we have an adequate sample size, giving us sufficient statistical power to explore the associations. Despite this, several limitations should be noted. The study was conducted using a convenience sample, relatively homogenous in age and health, thus the participants may not represent the general older population (in fact, the inclusion/exclusion criteria prevent, for instance, the inclusion of individuals with neuropathological/degenerative disease). This may lead to attenuation in correlations and can influence the strength or bias of the correlations among variables (Fabrigar et al., 1999) and, therefore, findings must be validated in future research in a larger non-selected sample with a variety of age-related chronic conditions allowing the generalization of the results. However, the study includes participants in living at home/community without asking for any change in their daily life activities, thus enabling to assess individuals who are often excluded from clinic-based studies. A further limitation to consider is the cross-sectional and observational nature of the study; therefore, any causal or temporal association between the levels of PA and brain health cannot be inferred. Therefore, the associations observed in this study need to be tested in longitudinal observational and/or interventional studies. Additionally, reverse causation must be considered, since individuals with better brain health may have a physically more active lifestyle. Important to mention is that although specifically ask to continue their normal routines, individuals could have modified their behavior, specifically in response to their awareness of being observed (Hawthorne effect). Although we performed the analysis between PA and brain health after controlling for age, gender, MMSE, and GDS, we did not consider APOE genotype. Since APOE ε4 allele carriage is considered the greatest non-modifiable risk factor for Alzheimer’s disease, this is an important confounding factor that should be evaluated in future studies, as a potential moderator of the relationship between PA and brain health (de Frutos-Lucas et al., 2020). In fact, other authors that classified the participants into four subgroups based on the PA level and presence of APOE-ε4 observed enhanced semantic memory processing in more physically active individuals, and this activation difference was most pronounced in those who possessed one or both APOE-ε4 alleles (Smith et al., 2011). Moreover, the protective effect of PA on hippocampal volume seems to be observed only in APOE-ε4 carriers (Smith et al., 2014). Regarding our 15-day monitoring protocol for PA assessment, it should be noted that this short period of time may not reflect the overall PA pattern of the participants and the seasonal variability. However, most of the studies evaluating PA often employed 7-day monitoring protocols. According to Hart et al. (2011) the continued use of a 7-day PA monitoring period is reliable in predicting PA and sedentary behavior. Moreover, a recent systematic review examining the association between PA and brain health has shown that usually PA monitoring periods range between 3 and 7 days (Domingos et al., 2021b). Finally, we observed an asymmetric distribution in the PA patterns, with the distribution being positively skewed. In fact, researchers in health and behavioral medicine, who study PA often use outcome variables that have a lower bound of zero and are positively skewed (Baldwin et al., 2016). This result makes sense because it is expected that most older adults have lower levels of PA, with just a few having higher levels. Therefore, we considered that the more extreme values observed in Figures 3, 4 are not necessarily outliers and are likely important values to explore the question at hand. Moreover, since we have a considerable sample size and used non-parametric methods (that have no assumptions on the data distribution) to establish the association with the MRI findings these values are not problematic, but important to address the question. Nonetheless, future studies using stratified random sampling with respect to PA levels could provide valuable insights into the relationship between PA and brain health. In conclusion, study findings suggest that the maintenance of hippocampal volume in healthy older adults is specifically associated with time spent in moderate and vigorous PA activities. Moreover, moderate PA and total PA time specifically increase brain FC in the frontal gyrus cingulate gyrus, parahippocampal gyrus, and in cerebellum occipital inferior lobe which are related with several cognitive functions, including memory, executive function, motor functions, visual and emotions processing. Altogether, the study provides new insights regarding the association between brain health and PA throughout the aging process.

## Data Availability Statement

The datasets generated for this study are available on request to the corresponding author.

## Ethics Statement

This study was conducted according to the Helsinki Declaration (59th Amendment), approved by the local ethical committees (Approval Number 42-2018), and the Portuguese Data Protection Authority (Approval Number 11286/2017) and developed in compliance with the new European General Data Protection Regulation. The study goals and assessments were explained during screening procedures. All participants provided written informed consent before study enrollment, which included consent to their data processing.

## Author Contributions

CD: conceptualization, data curation, formal analysis, investigation, methodology, writing – original draft, and writing – review and editing. MP-P: MRI formal analysis, MRI methodology, and writing – review and editing. RM: MRI formal analysis, MRI methodology, and writing – review and editing. MM: neuropsychological evaluations. NS: funding acquisition, supervision, and writing – review and editing. NCS: conceptualization, methodology, funding acquisition, supervision, and writing – review and editing. JMP: conceptualization, methodology, funding acquisition, supervision, writing – review and editing. All authors reviewed and approved the final version of the manuscript.

## Conflict of Interest

CD and JMP were employed by company iCognitus4ALL – IT Solutions. The remaining authors declare that the research was conducted in the absence of any commercial or financial relationships that could be construed as a potential conflict of interest.

## Publisher’s Note

All claims expressed in this article are solely those of the authors and do not necessarily represent those of their affiliated organizations, or those of the publisher, the editors and the reviewers. Any product that may be evaluated in this article, or claim that may be made by its manufacturer, is not guaranteed or endorsed by the publisher.

## Footnotes

1
https://osf.io/qepc8

## Supplementary Material

Supplementary Figure 1 Distribution of PA components (light, moderate and vigorous time) according to gender.

Supplementary Figure 2 Distribution of PA components (total PA and sedentary time) according to gender.

Supplementary Table 1 Spearman correlations between neuropsychological characteristics and PA components.
