# Supplementary figures and images for: Free-Living Physical Activity Measured With a Wearable Device Is Associated With Larger Hippocampus Volume and Greater Functional Connectivity in Healthy Older Adults: An Observational, Cross-Sectional Study in Northern Portugal
Source: Front Aging Neurosci. 2021 Nov 30;13:729060. doi: 10.3389/fnagi.2021.729060 (PMC8670087; doi:10.3389/fnagi.2021.729060)

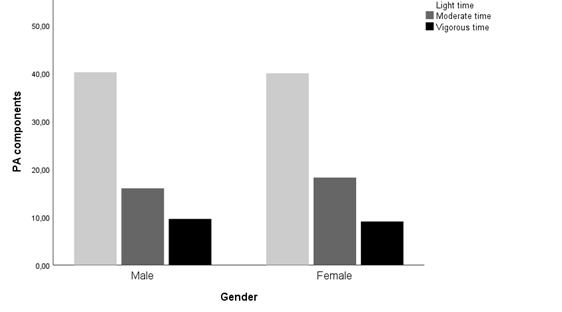

Supplement: Supplementary Figure 1 — Distribution of PA components (light, moderate and vigorous time) according to gender. [file Image_1.TIF]

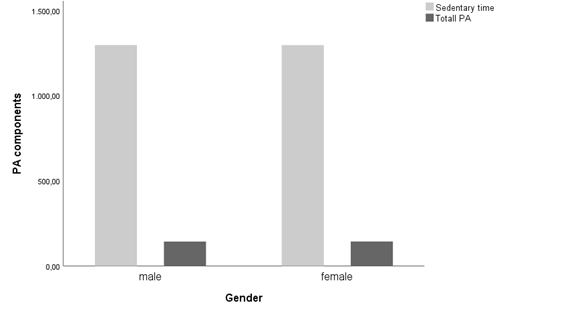

Supplement: Supplementary Figure 2 — Distribution of PA components (total PA and sedentary time) according to gender. [file Image_2.TIF]
