# Supplementary material for: Free-Living Physical Activity Measured With a Wearable Device Is Associated With Larger Hippocampus Volume and Greater Functional Connectivity in Healthy Older Adults: An Observational, Cross-Sectional Study in Northern Portugal
Source: Front Aging Neurosci. 2021 Nov 30;13:729060. doi: 10.3389/fnagi.2021.729060 (PMC8670087; doi:10.3389/fnagi.2021.729060)
Supplement: Supplementary Table 1 — Spearman correlations between neuropsychological characteristics and PA components. [file Table_1.DOCX]

**Supplementary Table 1.** Spearman correlations between neuropsychological characteristics and PA components.

| Neuropsychologic characteristics | Light time | Moderate time | Vigorous time | Total PA | Sedentary time | |
| --- | --- | --- | --- | --- | --- | --- |
| MMSE |  |  |  |  |  |  |
| r | -0.044 | 0.029 | 0.050 | 0.036 | -0.036 |  |
| *p*-value | 0.660 | 0.769 | 0.612 | 0.716 | 0.716 |  |
|  |  |  |  |  |  |  |
| GDS |  |  |  |  |  |  |
| r | 0.115 | 0.046 | -0.062 | 0.103 | -0.103 |  |
| *p*-value | 0.244 | 0.645 | 0.532 | 0.299 | 0.299 |  |
|  |  |  |  |  |  |  |
| SRT-LTS |  |  |  |  |  |  |
| r | -0.033 | 0.012 | -0.059 | 0.002 | -0.002 |  |
| *p*-value | 0.737 | 0.900 | 0.554 | 0.986 | 0.986 |  |
|  |  |  |  |  |  |  |
| SRT-CLTR |  |  |  |  |  |  |
| r | 0.001 | 0.026 | -0.116 | 0.003 | -0.003 |  |
| *p*-value | 0.991 | 0.790 | 0.240 | 0.972 | 0.972 |  |
|  |  |  |  |  |  |  |
| SRT-DR |  |  |  |  |  |  |
| r | 0.018 | 0.055 | 0.010 | 0.053 | -0.053 |  |
| *p*-value | 0.853 | 0.576 | 0.919 | 0.590 | 0.590 |  |
|  |  |  |  |  |  |  |
| Stroop-golden |  |  |  |  |  |  |
| r | 0.055 | 0.058 | 0.022 | 0.094 | -0.094 |  |
| *p*-value | 0.579 | 0.559 | 0.825 | 0.346 | 0.346 |  |
|  |  |  |  |  |  |  |
| DSST score |  |  |  |  |  |  |
| r | 0.089 | 0.196^*^ | 0.066 | 0.114 | -0.114 |  |
| *p*-value | 0.370 | 0.046 | 0.508 | 0.248 | 0.248 |  |
|  |  |  |  |  |  |  |
| Digits Span Test |  |  |  |  |  |  |
| r | -0.062 | -0.073 | -0.111 | 0.034 | -0.034 |  |
| *p*-value | 0.533 | 0.463 | 0.263 | 0.729 | 0.729 |  |
|  |  |  |  |  |  |  |
| COWAT FAS Admissible |  |  |  |  |  |  |
| r | -0.040 | 0.131 | 0.078 | 0.021 | -0.021 |  |
| *p*-value | 0.689 | 0.184 | 0.431 | 0.832 | 0.832 |  |
